# Effect of nicotinamide riboside on lipid metabolism and gut microflora‐bile acid axis in alcohol‐exposed mice

**DOI:** 10.1002/fsn3.2007

**Published:** 2020-12-21

**Authors:** Xiao Yu, Meilan Xue, Ying Liu, Zhitong Zhou, Yushan Jiang, Ting Sun, Hui Liang

**Affiliations:** ^1^ Department of Human Nutrition College of Public Health Qingdao University Qingdao China; ^2^ Basic Medical College Qingdao University of Medicine Qingdao China; ^3^ Food Science Department University of Guelph Guelph ON Canada

**Keywords:** alcoholic liver disease, gut microflora‐bile acid axis, lipid metabolism, nicotinamide riboside

## Abstract

Alcoholic liver disease (ALD) is the most common complication of alcohol abuse, while we lack safe and effective treatment for ALD. This study aimed to explore the effects of nicotinamide riboside (NR) on lipid metabolism and gut microflora‐bile acid axis in alcohol‐exposed mice. NR significantly improved liver histopathological damage and abnormal liver function. NR as a provider of nicotinamide adenine dinucleotide (NAD+) increased the NAD+/NADH ratio. Meanwhile, NR inhibited the activation of the protein phosphatase 1 signaling pathway, decreased the liver triglyceride and total bile acid levels, and reduced lipid accumulation. According to the results of gut microflora species analysis, NR intervention changed the microbial community structure at the phylum, family and genus levels, and the species abundances returned to a level similar to these of the normal control group. Besides, the results of high‐performance liquid chromatograph‐mass spectrometry showed that NR intervention resulted in fecal bile acid levels tending to be normal with decreased chenodeoxycholic acid level and increased deoxycholic acid and hyocholic acid levels. Spearman's correlation analysis showed a correlation between gut microflora and bile acids. Therefore, NR supplementation has the potential to prevent ALD, and its mechanism may be related to regulating lipid metabolism disorders and the gut microflora‐bile acid axis.

## Graphical abstract

The protective effect of nicotinamide riboside (NR) on liver in alcohol‐exposed mice. NR supplementation could regulate lipid metabolism and the gut flora‐bile acid axis to protect mice from ALD.

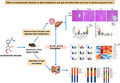

## 1 INTRODUCTION

Alcohol abuse is a severe global public health problem, which has become one of the top five risk factors for death and disability worldwide (Lim et al., 2012). According to the Global status report on alcohol and health 2018, alcohol abuse caused more than three million deaths (5.3% of all deaths) in 2016 (WHO, 2018). Alcoholic liver disease (ALD) is a progressive liver injury caused by chronic alcohol consumption, with a significant dose‐response relationship between the severity of ALD and alcohol consumption (Mathurin & Bataller, 2015). Lipid metabolism disorder is the early manifestation of ALD, with reversibility. Therefore, early regulation of lipid metabolism has essential significance for ALD treatment (Shen et al., 2019; Yang et al., 2017). Studies have shown that gut microflora is closely related to lipid metabolism (Chu et al., 2019; Le Roy et al., 2013), especially the bidirectional regulation with bile acids. Alcohol consumption could cause the imbalance of gut microflora and destroy the homeostasis of bile acid metabolism. Recently, the critical role of gut microflora and bile acids in ALD has been demonstrated (Hartmann et al., 2018; Panyod et al., 2020). Moreover, Wahlstrom et al. (Wahlström et al., 2017) have confirmed the concept of gut microflora‐bile acid axis. Therefore, the regulation of the gut microflora‐bile acid axis provides a new therapeutic approach for ALD.

Nicotinamide riboside (NR), the third component of Vitamin B3 (VB3) complex existed in milk and yeast, is considered to be a crucial nutritional precursor of nicotinamide adenine dinucleotide (NAD+) and its phosphorylated form (NADP+) (Bieganowski & Brenner, 2004). It should be noted that NAD+ precursors effectively improve neuropathy, steatosis, and inflammation in animal experiments (Hamity et al., 2017; Katsyuba et al., 2018; Yamamoto et al., 2014), which proves the feasibility of disease treatment by NR supplement. Emerging evidence suggested that NR could prevent ALD by reducing oxidative stress and activating SIRT1‐PGC‐1α‐mitochondrial biosynthesis (Wang et al., 2018). Our previous study found that dietary supplementation of NR protects against alcohol‐induced depression‐like behaviors by altering the composition of the gut microflora (Jiang et al., 2020). However, the potential effects of NR on lipid metabolism and gut microflora‐bile acid axis in alcohol‐exposed mice have not been explored. Therefore, our study was aimed to investigate the protective effects of NR on alcohol‐exposed mice via lipid metabolism and gut microflora‐bile acid axis.

## 2 MATERIALS AND METHODS

### 2.1 Experimental animals and ethics statement

Eight‐week‐old specific pathogen‐free (SPF) male C57BL/6J mice, weighing 22 ± 2 g, were obtained from Beijing Vital River Laboratory Animal Technology Co., Ltd (Beijing, China). Mice were housed in a light‐controlled room (12 hr light‐dark cycle) with controlled temperature (21 ± 2°C) and relative humidity (50%–60%). During the whole experiment, mice have free access to rodent chow and tap water ad libitum. This study was conducted following the National Institutes of Health Guide for Care and Use of Laboratory Animals (Publication No. 85‐23, revised 1985). The Review Committee for the Use of Human or Animal Subjects of the Medical College of Qingdao approved all experimental procedures.

### 2.2 Animal treatment and experimental design

After acclimatization for 1 week, mice were randomly divided into 4 groups (*n* = 10): the normal control group (NC), gavaged with normal saline; the ethanol model group (Model), gavaged with 50% (v/v) ethanol 8 ml/(kg.bw.d) for 2 weeks + 12 ml/(kg.bw.d) for 6 weeks; the NR intervention group (NR), gavaged with NR 400 mg/(kg.bw.d) + ethanol; the positive control group (PC), gavaged with diammonium glycyrrhizinate 200 mg/(kg.bw.d) + ethanol. The latter two groups were given ethanol at the same dose as the Model group after intervention treatment for 1 hr, while the other groups were given equivalent normal saline at the same time. Dose levels of NR and diammonium glycyrrhizinate were selected according to previous studies (Gao et al., 2019; Wang et al., 2018). Only one mouse was kept in each cage during the experiment. NR powder was purchased from Hubei Jusheng Technology Co., Ltd. and dissolved in normal saline at 80 mg/ml. Body weight was recorded weekly to adjust injection doses.

At the end of the 8‐week experiment, mice were fasted for 12 hr after the last gavage. Then, fecal samples were collected into 5 ml dry sterile Eppendorf tubes. Blood samples were collected by removing eyeballs and centrifuged at 827 g for 10 min to obtain serum. Fresh liver tissues were rapidly dissected and rinsed with iced‐cooled saline. Fecal samples, serum, and liver tissues were stored at −80°C for further analyses.

### 2.3 Liver histopathological analysis

The pathological changes of liver were observed by hematoxylin and eosin (H&E) staining. Liver tissues of 1.0 cm^3^ from four groups were fixed with 10% neutral formalin and embedded in paraffin. The paraffin‐embedded tissues were cut into 5 μm with a sliding microtome (Sakura TTM‐200‐NO) and dewaxed in xylene. After staining with H&E, the pathological changes of tissue slices were observed by a light microscope (Olympus BX60, Japan). Liver pathology was scored as described by Nanji et al. (Nanji et al., 1989), and the results were statistically analyzed. An experimenter and a rodent pathologist evaluated the pathological changes of liver tissues by the double‐blind method.

The ultrastructural changes of hepatocytes were observed by transmission electron microscopy (TEM). Liver tissues of 1.0 mm^3^ from four groups were fixed with 3% glutaraldehyde for 4 hr and rinsed with 0.1 mol/L phosphate buffer. Then tissues were fixed with 1% osmium tetroxide for 1 hr and rinsed three times. After dehydration with graded acetone, tissues were embedded in EPON812 and solidified in a heated incubator. Then, tissues were cut into 50–70 nm with an ultramicrotome (LKB, Stockholm, Sweden) and stained with 3% uranyl acetate and lead citrate to observe the pathological changes of hepatocytes by a JEM‐1200EX TEM (JEOL).

### 2.4 Determination of transaminase activities

This study was conducted to determine serum alanine aminotransferase (ALT) and aspartate aminotransferase (AST) activities by commercial assay kits (Nanjing Jiancheng Bioengineering Corp) according to the manufacturers' instructions. The method of serum treatment has been supplemented in the experimental design section. The attenuation rate of absorbance was determined by an automatic biochemical analyzer at 340 nm wavelength. The activities of ALT and AST were calculated according to the formula provided in the instructions.

### 2.5 Determination of liver NAD+ and NADH levels

This study was conducted to determine liver NAD+ and reducing equivalent (NADH) levels by commercial assay kits (Nanjing Jiancheng) according to the manufacturers' instructions. Liver tissues were washed twice with phosphate‐buffered saline. Liver homogenate was centrifuged at 827 g for 10 min at 4°C to collect the supernatant. The absorbance was determined by a microplate analyzer at 450 nm wavelength. The NAD+ and NADH levels in liver samples were calculated by the standard curve, and then, the ratio of NAD+/NADH was calculated.

### 2.6 Determination of serum and liver lipid levels

This study was conducted to determine serum and liver triglyceride (TG), total cholesterol (TC), low‐density lipoprotein cholesterol (LDL‐C), high‐density lipoprotein cholesterol (HDL‐C), and total bile acid (TBA) levels by commercial assay kits (Nanjing Jiancheng) according to the manufacturers' instructions. Liver total lipids were extracted by a chloroform/methanol mixture (2:1, v/v) at 827 g for 5 min, and then, the extracts were evaporated and redissolved with Triton X‐100. The absorbance of TG, TC, LDL‐C, HDL‐C, and TBA in serum and liver were determined at 510 nm, 510 nm, 546 nm, 546 nm, and 405 nm wavelength, respectively. Then, the levels of the above indicators were calculated according to the formula provided in the instructions.

### 2.7 Western blotting

Liver tissues were homogenized with ice‐cold radio immunoprecipitation assay (RIPA) buffer (Beyotime). Protein was quantified with a bicinchoninic acid (BCA) protein assay kit (Beyotime). Equal amounts of protein were separated by 10% sodium dodecyl sulfate‐polyacrylamide gel electrophoresis (SDS‐PAGE) for 2 hr and transferred to polyvinylidene fluoride (PVDF) membranes (Millipore) for 1 hr. After blocked with 5% skim milk powder for 2 hr at room temperature, membranes were incubated with primary antibodies overnight at 4°C. The antibodies against phosphatase 1 α (PP1α), p‐DNA‐dependent protein kinase (p‐DNA‐PK), upstream stimulatory factor 1 (USF1), and fatty acid synthase (FAS) were purchased from Santa Cruz Biotechnology, Inc. After washed three times with Tris‐buffered saline (TBS) solution, membranes were incubated with corresponding secondary antibodies. Afterward, protein bands were visualized with enhanced chemiluminescence (ECL, Perkin Elmer). β‐actin was used as an internal control to assess protein loading in each lane.

### 2.8 Fecal bile acid analysis

Fecal samples were operated based on the previous study (Shi et al., 2016). In short, fecal samples were soaked with methanol, then extracted by ultrasonication and centrifuged. After filtration with 0.45 μm membranes, removed samples of 10 μl were analyzed using the high‐performance liquid chromatograph‐mass spectrometer (HPLC‐MS, Agilent Technologies).

### 2.9 16S rDNA high‐throughput sequencing

After 8 weeks of intervention, the 16S rDNA gene sequencing was performed on fecal samples from the NC group, Model group and NR group (*n* = 5 in each group), according to our previous study (Jiang et al., 2020). Briefly, the QIAamp Fast DNA Stool Mini Kit (QIAGEN) was used to extract genomic DNA from frozen fecal samples according to the manufacturers' instructions. Then 0.8% agarose gel electrophoresis was used to detect DNA concentration and purity. The V3‐V4 regions of the 16S rDNA gene were amplified with the general primers (F341: CCTACGGGRSGCAGCAG, R806: GGACTACVVGGGTATCTAATC). The 16S rDNA gene sequencing was entrusted to the Realbio Genomics Institute (Shanghai, China) by using the Illumina HiSeq PE250 sequencing platform (Illumina, Inc.), and the analysis was carried out by Ruiyi Biotechnologies Inc.

### 2.10 Statistical analysis

Data were analyzed using SPSS 22.0 (SPSS) and GraphPad Prism 8.0 (GraphPad Software). Data were expressed as means ± standard deviation (*SD*). Comparisons among multiple groups were analyzed by one‐way analysis of variance (ANOVA), followed by Fisher's least significant difference (LSD) post hoc test. Correlation analysis between gut microflora and bile acids was identified using Spearman's rank correlation, and the heatmap analysis was performed using R3.1.0. *p* < .05 was considered as statistically significant.

## 3 RESULTS

### 3.1 Effect of NR on body weight

**FIGURE 1:**
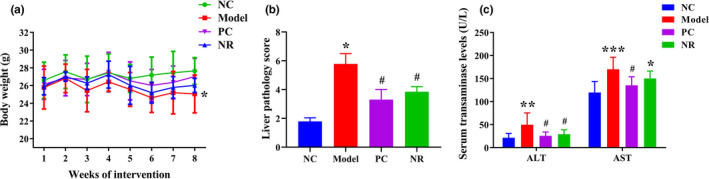
NR intervention improved body weight, histopathological damage, and serum aminotransferase activities. (a) Body weight of mice. (b) The pathological score of liver tissues. (c) Serum aminotransferase activities. Values are expressed as means ± *SD*. *p < .05 versus NC; **p < .01 versus NC; ***p < .001 versus NC; #*p* < .05 versus Model. NC, the normal control group; Model, the ethanol model group; NR, the NR intervention group

Alcohol intake has a toxic effect on mice, which in turn affects their growing development. As shown in Figure 1a, after 8 weeks of intervention, the body weight of mice in the Model group was lower than that of the NC group (*p* < .05). Inversely, compared with the Model group, the body weight in the NR and PC groups were increased in a degree, but the difference was not statistically significant (*p* > .05).

### 3.2 Effect of NR on liver histopathological changes

**FIGURE 2:**
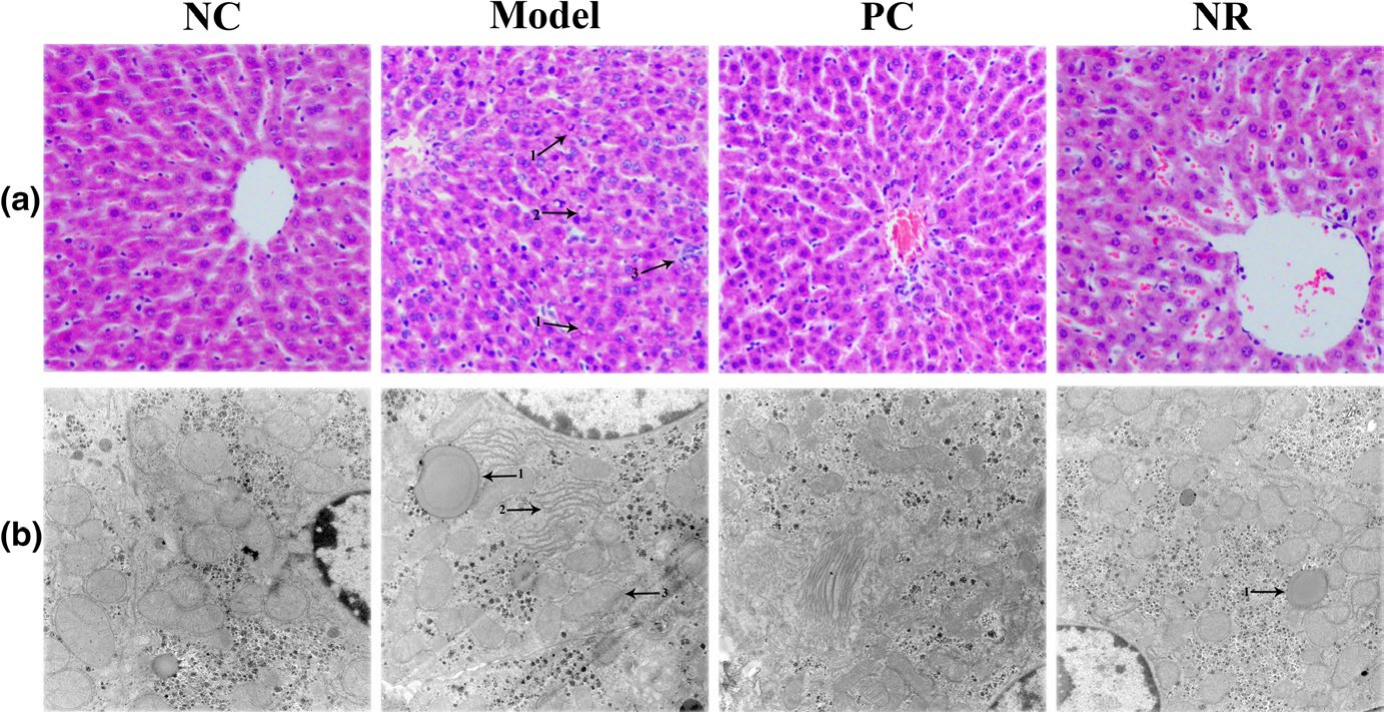
Effect of NR on histopathological changes in mice liver tissues. (a) The histological changes of liver sections stained with H&E (200×). Arrow 1, lipid droplet; arrow 2, Mallory body; arrow 3, inflammatory cell infiltration. (b) The ultrastructure changes in liver cells were observed by TEM (20,000×). Arrow 1, lipid droplet; arrow 2, endoplasmic reticulum; arrow 3, mitochondrion. NC, the normal control group; Model, the ethanol model group; NR, the NR intervention group

H&E staining results are shown in Figure 2a. The liver lobule structure was normal in the NC group mice, liver cells were arranged neatly without steatosis, and the nuclei were round. Mice in the Model group showed apparent pathological damage, including liver cell swelling, steatosis, cytoplasmic cavitation, and inflammatory cell infiltration. Simultaneously, Mallory bodies of different shapes were visible in the Model group, and the pathological score was significantly higher than that in the NC group (Figure 1b) (*p* < .05). However, those pathological changes were alleviated considerably after NR and diammonium glycyrrhizinate intervention, and the liver pathology score was decreased compared with that in the Model group (*p* < .05).

Transmission electron microscopy observation results are shown in Figure 2b. Liver cells in the NC group were in good condition, with abundant organelles and few abnormal changes. Alcohol exposure caused changes in the structure of liver cells. In the Model group, lipid droplets were observed, the number of mitochondria was reduced, the ridge was fuzzy, and the endoplasmic reticulum was degranulated. However, liver cell damage was significantly improved in the NR and PC groups.

### 3.3 Effect of NR on serum aminotransferase

Serum transaminase activities are shown in Figure 1c. In the Model group, serum ALT and AST activities were higher than those in the NC group (*p* < .01), while NR and diammonium glycyrrhizinate intervention could reverse the above changes. The activities of ALT and AST in the PC group and the activity of ALT in the NR group were significantly decreased when compared with those in the Model group (*p* < .05).

### 3.4 Effect of NR on liver NAD+ and NADH levels

**FIGURE 3:**
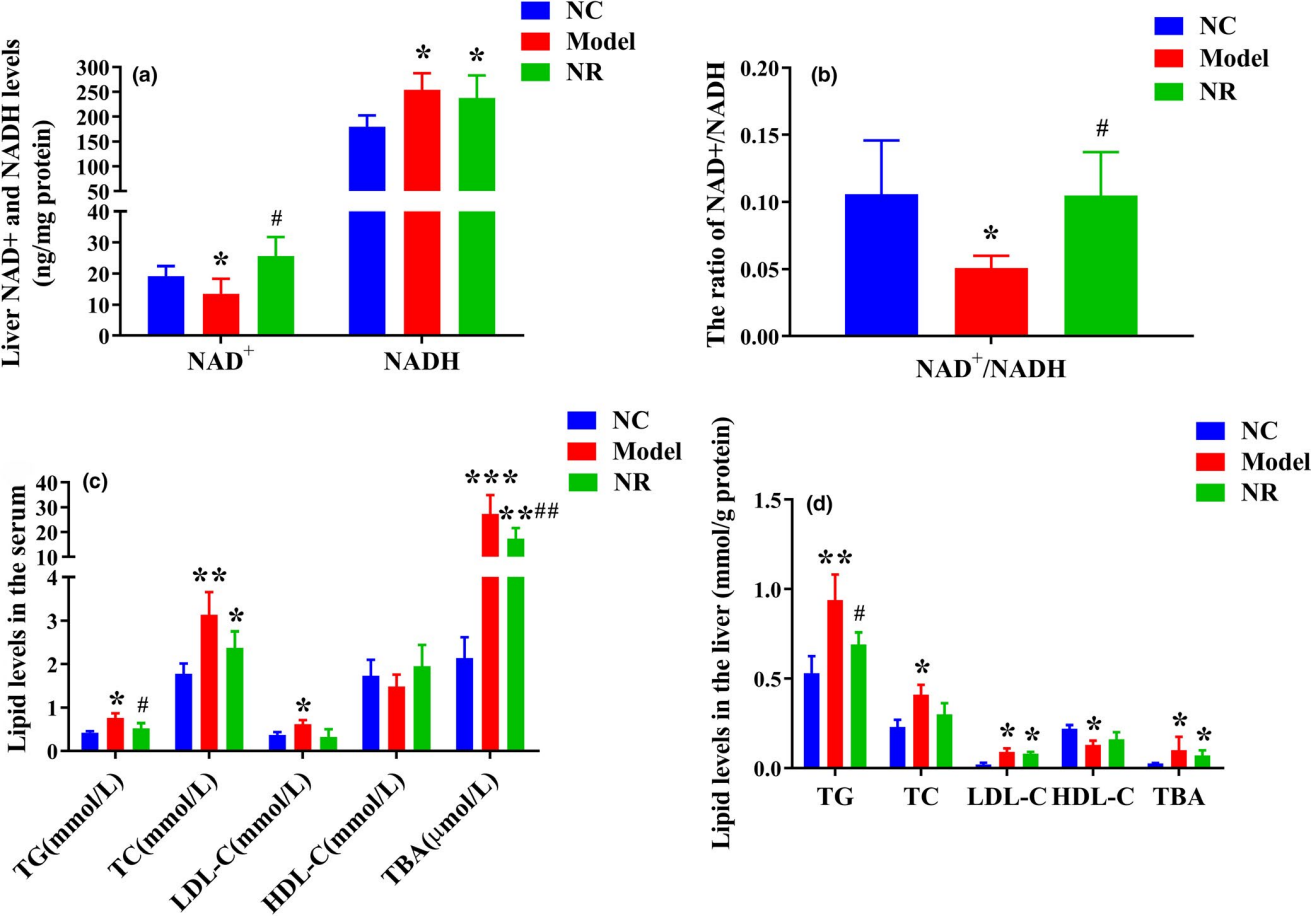
NR intervention increased the NAD^+^/NADH ratio and regulated lipid metabolism disorder. (a) Liver NAD^+^ and NADH levels. (b) Liver NAD^+^/NADH ratio. (c) Serum TG, TC, LDL‐C, HDL‐C and TBA levels. (d) Liver TG, TC, LDL‐C, HDL‐C and TBA levels. Values are expressed as means ± *SD*. *p < .05 versus NC; **p < .01 versus NC; ***p < .001 versus NC; #*p* < .05 versus Model; ##*p* < .01 versus Model. NC, the normal control group; Model, the ethanol model group; NR, the NR intervention group. NAD^+^, nicotinamide adenine dinucleotide; NADH, reducing equivalent; TG, liver triglyceride; TC, total cholesterol; LDL‐C, low‐density lipoprotein cholesterol; HDL‐C, high‐density lipoprotein cholesterol; TBA, total bile acid

Compared with the NC group, alcohol exposure significantly reduced the NAD+ level, increased the NADH level, and reduced the NAD+/NADH ratio (*p* < .05) (Figure 3a,b). However, NR intervention reversed the above changes, and the NAD+/NADH ratio was higher than that in the Model group (*p* < .05).

### 3.5 Effect of NR on lipid levels

Serum TG, TC, LDL‐C, and TBA levels in the Model group were significantly higher than those in the NC group (*p* < .05) (Figure 3c). Simultaneously, alcohol exposure resulted in lipid accumulation in liver. Liver TG, TC, LDL‐C, and TBA levels were elevated (*p* < .05) and HDL‐C level was reduced (*p* < .05) when compared with those in the NC group (Figure 3d). However, NR intervention inhibited lipid accumulation in serum and liver, especially serum TG and TBA levels and liver TG level were significantly decreased (*p* < .05).

### 3.6 Effect of NR on the liver PP1 signaling pathway

**FIGURE 4:**
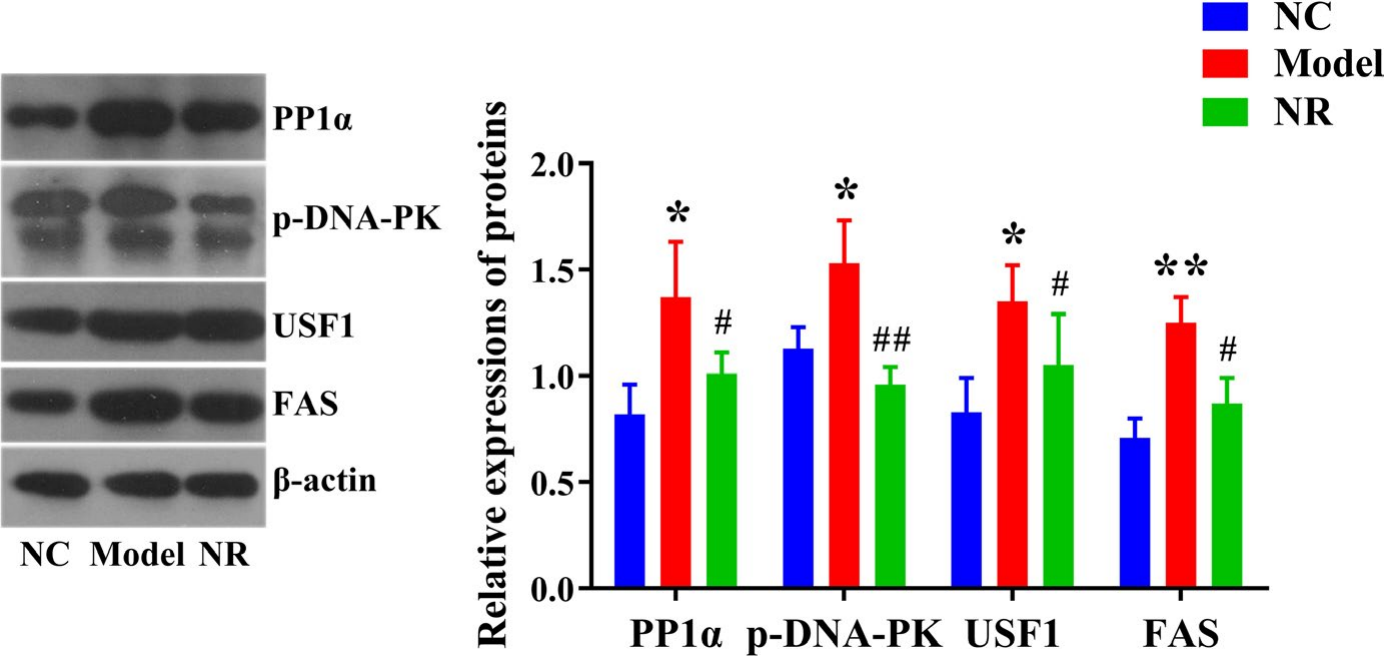
Effect of NR on the liver PP1 signaling pathway. Western blotting was used to analyze the expression of the PP1 signaling pathway in the liver. Values are expressed as means ± *SD*. *p < .05 versus NC; **p < .01 versus NC; #*p* < .05 versus Model; ##*p* < .01 versus Model. NC, the normal control group; Model, the ethanol model group; NR, the NR intervention group. PP1α, protein phosphatase 1α; p‐DNA‐PK, p‐DNA‐dependent protein kinase; USF1, upstream stimulatory factor 1; FAS, fatty acid synthase

Western blotting results showed that alcohol exposure activated the PP1 signaling pathway in the liver. Compared with the NC group, the expression levels of PP1α, p‐DNA‐PK, USF1, and FAS were significantly up‐regulated in the Model group (*p* < .05) (Figure 4). However, NR intervention could inhibit the activation of the PP1 signaling pathway. The expression levels of PP1α, p‐DNA‐PK, USF1, and FAS in the NR group were significantly lower than those in the Model group (*p* < .05).

### 3.7 Effect of NR on fecal bile acid levels

**FIGURE 5:**
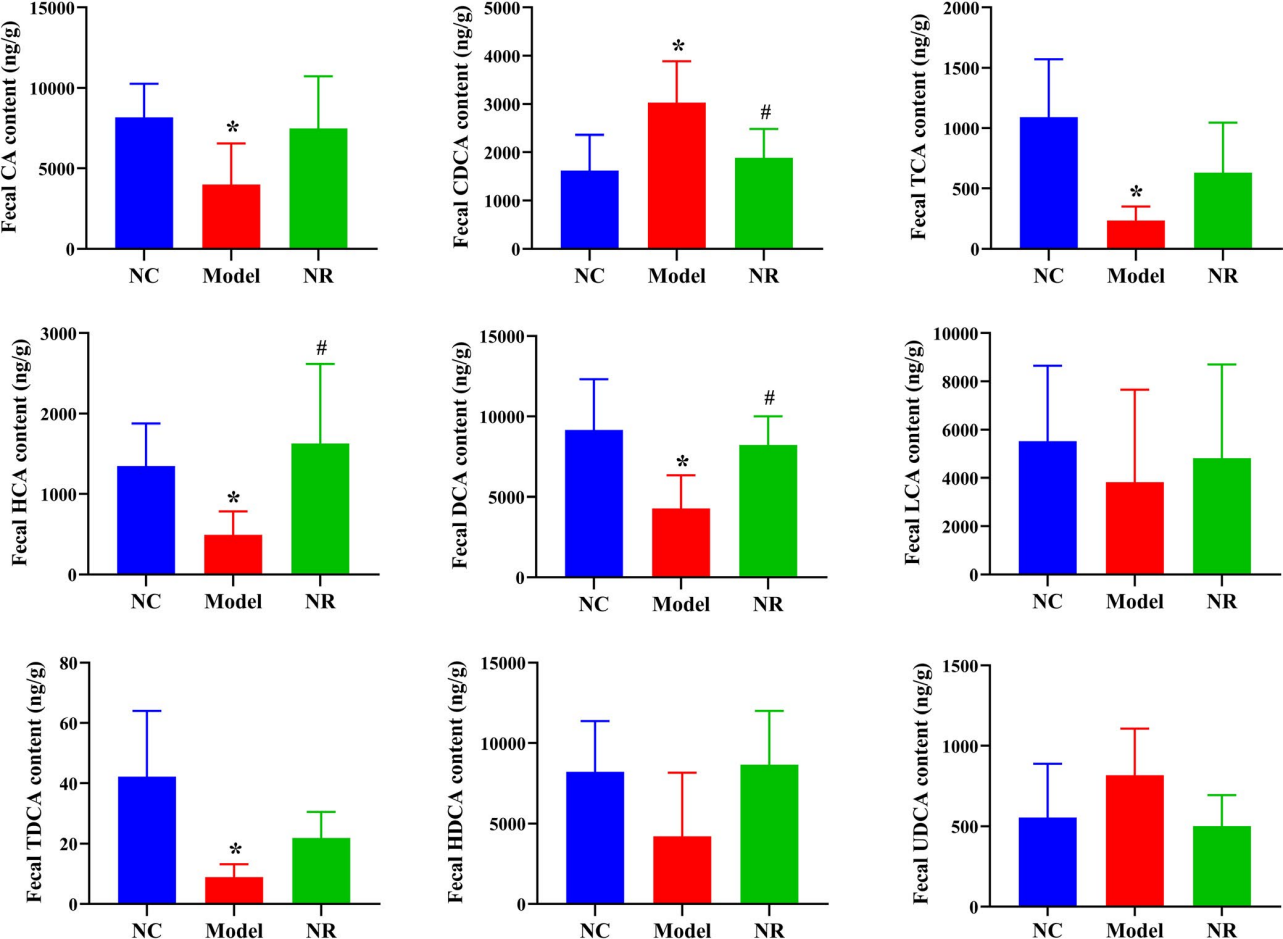
Effect of NR on fecal bile acid levels. A simple HPLC‐MS method was used to quantitate fecal bile acids. Values are expressed as means ± *SD*. *p < .05 versus NC; #*p* < .05 versus Model. NC, the normal control group; Model, the ethanol model group; NR, the NR intervention group. CA, Cholic acid; CDCA, Chenodesoxycholic acid; TCA, Taurocholic acid; HCA, Hyocholic acid; DCA, Deoxycholic acid; LCA, Lithocholic acid; TDCA, Taurodeoxycholic acid; HDCA, Hyodeoxycholic acid; UDCA, Ursodeoxycholic acid

The bile acid chromatograms of standard samples and fecal samples analyzed by HPLC‐MS were shown in Figures S1 and S2. Figure 5 showed the levels of fecal bile acids. In the Model group, fecal primary bile acids such as cholic acid (CA), taurocholic acid (TCA), hyocholic acid (HCA) levels, and secondary bile acids such as deoxycholic acid (DCA), taurodeoxycholic acid (TDCA) levels were reduced when compared with those in the NC group (*p* < .05), while chenodeoxycholic acid (CDCA) level was elevated (*p* < .05). After NR intervention, bile acid levels tended to be normal, especially CDCA level was decreased, DCA and HCA levels were increased when compared with those in the Model group (*p* < .05), and other bile acid levels were shown to decrease gradually.

### 3.8 16S rDNA high‐throughput sequencing analysis

**FIGURE 6:**
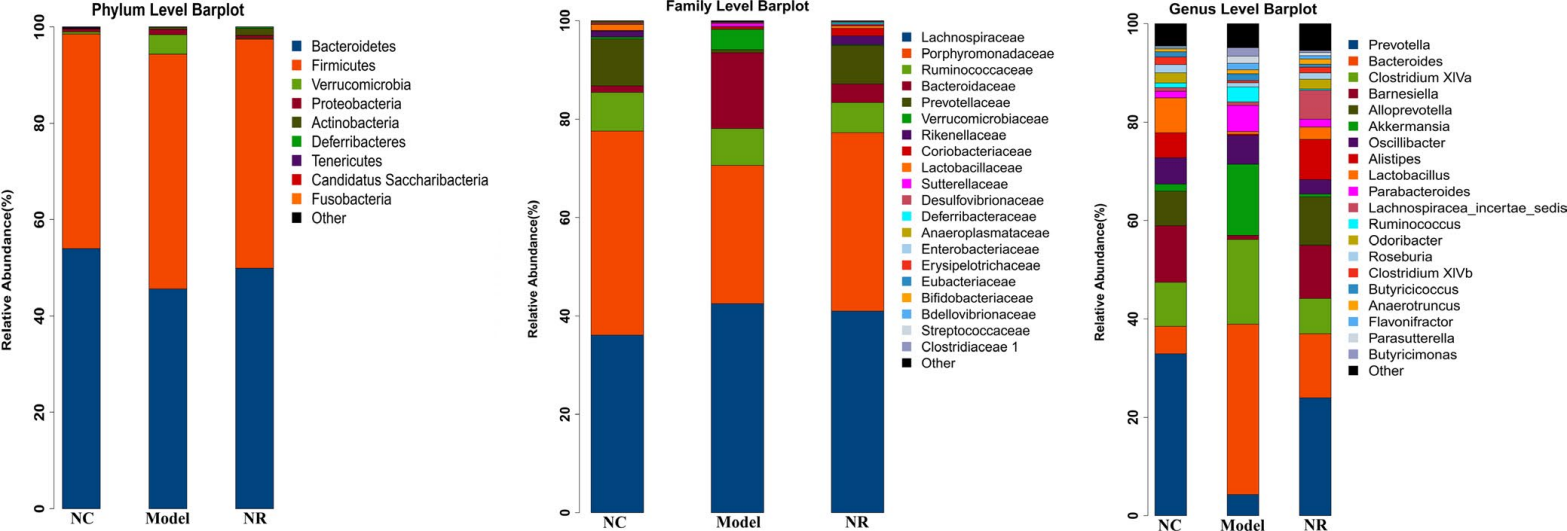
Effect of NR on the microbial community structure at the phylum, family and genus levels. The molecular ecology of fecal microflora was analyzed by 16S rDNA high‐throughput sequencing. NC, the normal control group; Model, the ethanol model group; NR, the NR intervention group

The molecular ecology of fecal microflora was analyzed by 16S rDNA high‐throughput sequencing. According to the species analysis results, the microbial community structure in the Model group was different from those in the NC and NR groups (Figure 6). The abundance of species at the phylum, family, and genus level in three groups were shown in Table 1.

**Table 1:** Species abundance analysis at phylum, family, and genus levels

Species‐level	Abundance (%)		
	NC	Model	NR
Phylum			
*Bacteroidetes*	54.00 ± 17.93	45.64 ± 7.85	49.94 ± 9.81
*Firmicutes*	44.56 ± 18.34	48.73 ± 10.54	47.53 ± 11.72
*Proteobacteria*	0.60 ± 0.52	1.06 ± 1.19	0.62 ± 0.28
*Actinobacteria*	0.11 ± 0.05	0.42 ± 0.26	1.42 ± 0.99^#^
*Deferribacteres*	0.08 ± 0.03	0.00 ± 0.00	0.29 ± 0.22^#^
Family			
*Lachnospiraceae*	36.11 ± 17.28	42.47 ± 9.82	41.00 ± 11.58
*Porphyromonadaceae*	41.45 ± 19.30	28.15 ± 10.65	36.21 ± 11.63
*Bacteroidaceae*	1.35 ± 1.51	15.38 ± 18.22	3.79 ± 6.38
*Prevotellaceae*	9.56 ± 6.64	0.63 ± 0.41*	7.77 ± 2.99^#^
*Verrucomicrobiaceae*	0.39 ± 0.22	4.14 ± 3.58*	0.17 ± 0.09^#^
*Lactobacillaceae*	1.26 ± 1.70	0.12 ± 0.12	0.54 ± 0.73
Genus			
*Prevotella*	32.94 ± 22.52	4.27 ± 8.43	23.98 ± 15.29
*Bacteroides*	5.53 ± 5.45	34.65 ± 20.34*	13.03 ± 12.56
*Barnesiella*	11.54 ± 12.65	0.83 ± 1.39	10.84 ± 5.79^#^
*Akkermansia*	1.43 ± 2.94	14.49 ± 11.33	0.57 ± 0.97
*Alloprevotella*	7.03 ± 3.98	0.02 ± 0.01*	9.82 ± 5.42^##^
*Alistipes*	5.09 ± 3.47	0.22 ± 0.51	8.24 ± 7.39
*Odoribacter*	2.02 ± 1.92	0.00 ± 0.00	2.01 ± 1.88

Note

Values are expressed as means ± *SD*.

Abbreviations: Model, the ethanol model group; NC, the normal control group; NR, the NR intervention group.

* *p* < .05 versus NC.

^#^ *p* < .05 versus Model.

^##^ *p* < .01 versus Model.

At the phylum level, the top five microorganisms in the gut microbiota of three groups were *Bacteroidetes*, *Firmicutes*, *Verrucomicrobia*, *Proteobacteria*, and *Actinobacteria*. Among them, the abundances of *Bacteroidetes* and *Firmicutes* accounted for more than 90% of the total microflora, but there was no significant difference in their abundances among the three groups (*p* > .05). It should be noted that the abundance of *Actinobacteria* increased gradually in the Model and NR groups, and its abundance in the NR group was higher than that in the Model group (*p* < .05). In addition, the abundance of *Deferribacteres* was absent after alcohol exposure, while it increased to 0.29% after NR supplement (*p* < .05).

At the family level, *Lachnospiraceae* and *Porphyromonadaceae* were the most abundant families in each group. Meanwhile, the abundance of *Bacteroidaceae* was also enriched in the Model group, but there was no significant difference compared with those in the NC and NR groups (*p* > .05). Significantly, the abundance of *Prevotellaceae* was 0.63% in the Model group, which was lower than those in the NC and NR groups (*p* < .05). Instead, the abundance of *Verrucomicrobiaceae* was enriched in the Model group compared with that in the NC group (*p* < .05), while the change was significantly reversed by NR intervention (*p* < .05).

At the genus level, the composition of the top 20 dominant genera in the NC group was similar to that in the NR group, and *Prevotella* was the dominant genus. However, the microbial community structure in alcohol‐exposed mice is different from the other two groups. The abundance of *Bacteroides* in the Model group was higher than that in the NC group (*p* < .05), becoming the most abundant genus. In addition, *Akkermansia* abundance was enriched after alcohol exposure, while there was no significant difference compared with that in the NC group (*p* > .05). Interestingly, the abundances of *Barnesiella*, *Alloprevotella*, *Alistipes* and *Odoribacter* in the NC group were 11.54%, 7.03%, 5.09% and 2.02%, respectively, while their abundances were decreased sharply or even absent in the Model group. Inversely, there was an upward trend in the abundances of the above genus after NR supplement, especially the abundances of *Barnesiella* and *Alloprevotella* were significantly different from those in the Model group (*p* < .05).

### 3.9 The correlation analysis between gut microflora and bile acids

**FIGURE 7:**
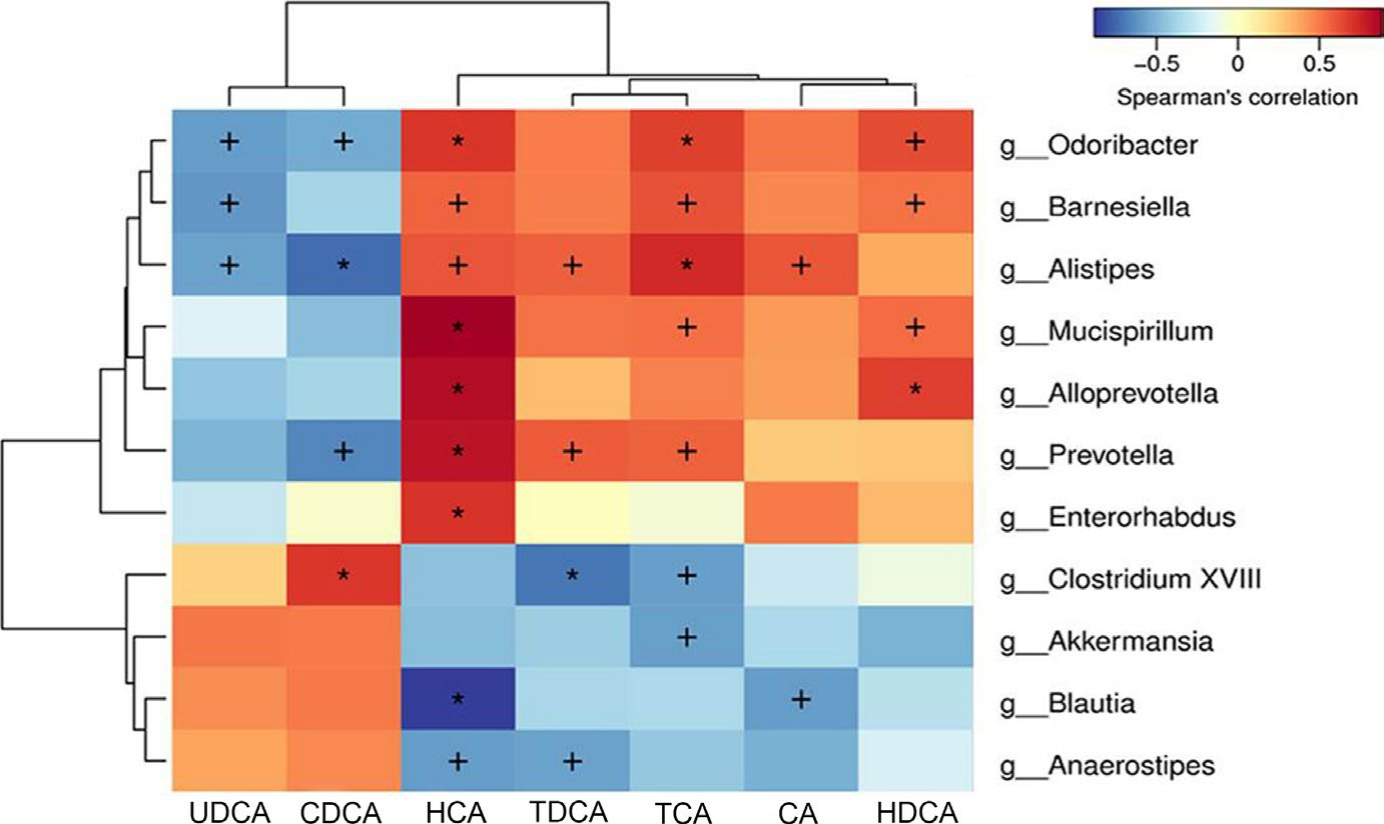
The correlation between gut microflora and bile acids. Spearman's rank correlation was adopted to analyze the correlation between gut microflora and bile acids. Pearson product difference correlation coefficients are expressed in blue (negative correlation [−1]) to red (positive correlation [+1]). +*p* < .05; *p < .01. UDCA, Ursodeoxycholic acid; CDCA, Chenodesoxycholic acid; HCA, Hyocholic acid; TDCA, Taurodeoxycholic acid; TCA, Taurocholic acid; CA, Cholic acid; HDCA, Hyodeoxycholic acid

As shown in Figure 7, Spearman's rank correlation analysis showed the correlation between gut microflora and bile acids. The abundances of *Odoribacter*, *Barnesiella*, and *Alistipes* were reduced or absent in the Model group and negatively correlated with ursodeoxycholic acid (UDCA) level. In addition, the abundances of *Odoribacter* and *Barnesiella* were positively correlated with HCA, TCA and hyodeoxycholic acid (HDCA) levels, and the abundance of *Alistipes* was positively correlated with HCA, TDCA, TCA and CA levels. The abundance of *Mucuspirillum* was positively correlated with HCA, TCA and HDCA levels. The abundance of *Prevotella* was negatively correlated with CDCA level, and positively correlated with HCA, TDCA and TCA levels. The abundance of *Clostridium XVIII* was negatively correlated with TDCA and TCA levels, and positively correlated with CDCA level.

## 4 DISCUSSION

It is well known that alcohol abuse severely harms human health and induces different degrees of liver injury. ALD is the most common complication of excessive drinking, and has become one of the most prevalent chronic liver diseases in China. Currently, we lack safe and effective treatment for ALD. We found that NR could effectively alleviate the liver injury caused by alcohol exposure, and its protective effect might be related to the regulation of lipid metabolism disorder and gut microflora‐bile acid axis. H&E staining and TEM observations revealed noticeable histopathological changes in the liver, such as steatosis, inflammatory cell infiltration, and mitochondria damage after alcohol exposure. Serum aminotransferases are sensitive indicators of liver injury (Huang et al., 2012). In our study, similar to the results of Zhang et al. (Zhang et al., 2017), alcohol exposure reduced the body weight of mice, which may be related to the toxic effects of alcohol on food digestion and absorption in mice. In addition, serum ALT and AST activities were increased, and liver function was damaged in the Model group. At the same time, NR intervention could improve alcohol‐induced liver dysfunction, which was also confirmed in the study of Wang et al. (Wang et al., 2018), suggesting the improvement effect on ALD.

The pathogenesis of ALD is complex. It is generally believed that excessive alcohol consumption reduces the NAD^+^/NADH ratio, promotes fatty acid synthesis, and affects liver lipid metabolism and blood lipid transport (Yoon et al., 2015). As expected, NR intervention significantly increased the NAD^+^/NADH ratio and improved serum and liver TG levels induced by alcohol. Evidence suggests that nicotinic acid could regulate alcohol‐induced lipid metabolism by increasing NAD^+^ level and then growing liver mitochondrial fatty acid β‐oxidation (Li et al., 2014), while the elevated NAD^+^ level in high‐fat diet mice could be directly synthesized by NR intervention (Cantó et al., 2012). Lima et al. (Lima et al., 2020) showed that NAD^+^ precursor is beneficial to steatolysis, resulting in tissue‐specific and adaptive lipid molecular changes in ovariectomized rats. Gariani et al. (Gariani et al., 2016) confirmed that ApoE^−^/^−^ mice with nonalcoholic fatty liver disease (NAFLD) could increase NAD^+^ level, reduce body weight and transaminase activities, and alleviate steatosis after NR intervention. These studies are similar to our results and provide a theoretical basis for improving lipid metabolism by supplementing NAD^+^ precursor. In addition, liver lipid homeostasis is strictly controlled by various mediators and enzymes. Sustained alcohol exposure activates PP1 and modifies phosphorylation of substrates associated with protein kinase A (Price et al., 2015). The important role of the PP1‐DNA‐PK‐USF1 signaling pathway in lipid metabolism has been confirmed (Casado et al., 1999; Kwan et al., 2013; Wang & He, 2018). Our results showed that NR intervention could inhibit the activation of the PP1‐DNA‐PK‐USF1 signaling pathway caused by alcohol exposure, thus inhibiting the occurrence of lipid metabolism disorders. The above results suggested the excellent effect of NR in improving ALD. As a provider of NAD^+^, NR could alleviate the lipid accumulation caused by alcohol exposure via inhibiting the activation of the PP1 pathway, promoting fatty acid oxidation, and inhibiting lipid synthesis.

In addition, we suggested that NR could improve alcohol‐induced liver injury by regulating the gut microflora‐bile acid axis. Gut microflora is considered as a "metabolic organ" and occupies a core position in the whole intestinal ecosystem, mainly composed of *Bifidobacteria*, *Firmicutes*, *Actinomycetes*, and *Bacteroides* (Chassaing et al., 2014). Alcohol consumption causes apparent changes in the composition of gut microflora leading to dysbiosis in animals and humans, directly affecting the occurrence of ALD and individual susceptibility to ALD (Ames et al., 2020; Llopis et al., 2016; Xue et al., 2017). In this study, we analyzed the species abundance at the phylum, family, and genus levels. The results showed that NR intervention changed the gut microflora structure in alcohol‐exposed mice and restored the abundances of gut microflora to a level similar to those in normal mice. At the phylum level, the majority of sequences belong to *Bacteroidetes* and *Firmicutes*, accounting for more than 90% of the total sequences, which is similar to the previous results (Wu et al., 2020), and play an essential role in body health. At the family level, *Lachnospiraceae* and *Porphyromonadaceae* were the most abundant. The abundance of *Actinobacteria* was increased significantly after NR intervention compared with that in the Model group. *Actinobacteria* is considered the primary producer of antibacterial drugs and produces abundant bioactive substances (Barka et al., 2016). In the gut, *Deferribacteres* is protective of the immune system (Ma et al., 2019), and *Verrucomicrobia* is believed to have nutritional benefits (Anhê et al., 2015). We found that NR intervention could reverse the absence of *Deferribacters* and the enrichment of *Verrucomicrobiaceae* (from *Verrucomicrobia*) induced by alcohol exposure. Similarly, this result was also confirmed in a high‐fructose intake model in mice (Wang et al., 2020), which was considered to be related to glucolipid metabolism. As mentioned earlier, our data also proved the regulatory effect of NR on lipid metabolism. In addition, compared with the Model group, NR intervention improved the abundance of *Prevotellaceae* at the family level and the abundances of *Barnesiella* and *Alloprevotella* at the genus level, and other species composition also changed to a degree. *Barnesiella* belongs to *Porphyromonadaceae*, and its obligate anaerobe could remove vancomycin‐resistant Enterococcus colonized in the intestinal and prevent the spread of highly resistant bacteria (Ubeda et al., 2013). The increase in *Prevotellaceae* was associated with the high expression of genes in specific metabolic processes (Gomez et al., 2016). *Alloprevotella* is from *Prevotellaceae*, which is negatively correlated with NAFLD progression (Shang et al., 2017), and could produce short‐chain fatty acids to play an anti‐inflammatory role (Ren et al., 2016). Both of them played a beneficial role in restoring the diversity of gut microflora and reducing the serum TC level (Bordoni et al., 2013; Gomez et al., 2016).

Bile acids are synthesized from cholesterol and further metabolized into secondary bile acids through gut microflora, which promotes the digestion and absorption of lipids, and are related to the metabolism of TG and TC (Chen et al., 2016). Lieber et al. (Lefevre et al., 1972) found for the first time that chronic alcohol consumption enlarged the bile acid pool and reduced the excretion of bile acid, which suggested that alcohol consumption could affect the enterohepatic circulation of bile acid. In addition, the signal of bile acid homeostasis can be sensed by the liver and intestine, which is the key to the homeostasis regulation of gut microflora (Li et al., 2019). In this study, alcohol exposure caused gut microflora and liver lipid metabolism disorder, leading to TG, TC, and TBA accumulation, probably caused by reduced excretion of fecal bile acids and increased intestinal reabsorption of bile acids. Our results of fecal bile acid level analysis showed that NR intervention restored the bile acid levels in mice feces to approximately normal levels, especially the increase of HCA and DCA. In addition, we evaluated the correlation between bile acids and the identified bacteria. It should be noted that HCA, TCA, and HDCA levels were positively correlated with most of the bacteria genera with reduced relative abundance after alcohol treatment, such as *Odoribacter*, *Barnesiella* and *Mucispirillum* and *Alloprevotella*. However, the bile acids levels and gut microflora composition tended to be normal after NR intervention, thereby protecting mice from ALD damage. Similar to our results, an experiment in mice (Out et al., 2015) showed that the reduction of gut microflora increased the intestinal bile acid reabsorption and significantly reduced the fecal bile acid levels. Additionally, Ciocan et al. (Ciocan et al., 2018) also proved that fecal TBA and secondary bile acid levels are lower in patients with severe alcoholic cirrhosis than those in nonalcoholic cirrhosis, which may be a vicious cycle caused by specific changes in gut microflora. These results confirmed that liver disease, bile acids, and gut microflora are important concepts, and the gut microflora‐bile acid axis is of great significance for the study of the pathogenesis of ALD. Differently, NR intervention significantly reduced the increase of CDCA level in mice feces induced by alcohol and was negatively correlated with the abundances of *Odoribacter*, *Alistipes*, and *Prevotella*. The alternative pathway of bile acid synthesis mainly generates CDCA, while the classical pathway simultaneously generates CDCA and CA. Sterol 12a‐hydroxylase (CYP8B1) is an essential enzyme for the production of CA, which catalyzes the conversion of CDCA to CA and determines the ratio of them (Li‐Hawkins et al., 2002). Therefore, we speculate that the increase of CDCA level and the decrease of CA level in the Model group were caused by the absence of CYP8B1 in the classical pathway of bile acids synthesis. However, whether the reduction of CDCA level after NR intervention could be the molecular mechanism for NR to protect ALD remains to be further clarified.

## 5 CONCLUSIONS

In summary, NR supplementation could increase the NAD+ level and has a protective effect on alcohol‐induced liver injury. The mechanism may be related to regulating lipid metabolism disorders and the gut microflora‐bile acid axis. NR is a novel component of VB3 complex and an important nutrient, then further studies of its mechanism could supply new bases for clinical treatment of ALD.

## CONFLICT OF INTEREST

The authors declare that they do not have any conflict of interest.

## ETHICAL APPROVAL

This study was approved by the Institutional Review Board of Human or Animal Subjects of the Medical College of Qingdao University.

## Supporting information

Fig S1

Fig S2

## Data Availability

All data supporting the conclusions of this study are included in this published article.
