# Supplementary figures and images for: Effect of nicotinamide riboside on lipid metabolism and gut microflora‐bile acid axis in alcohol‐exposed mice
Source: Food Sci Nutr. 2020 Dec 21;9(1):429–40. doi: 10.1002/fsn3.2007 (PMC7802554; doi:10.1002/fsn3.2007)

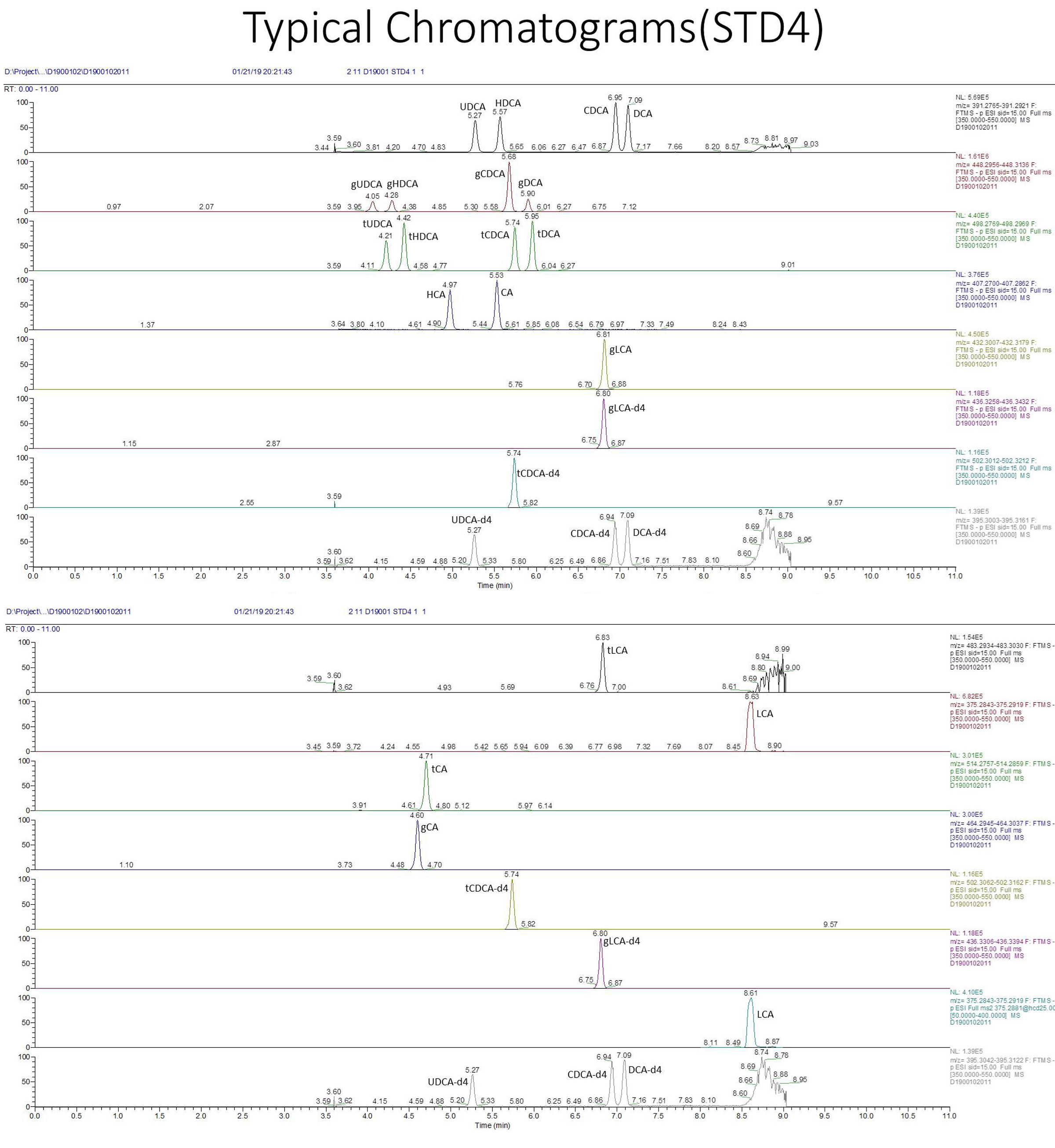

Supplement: Supplementary file 1 — Fig S1 [file FSN3-9-429-s001.jpg]

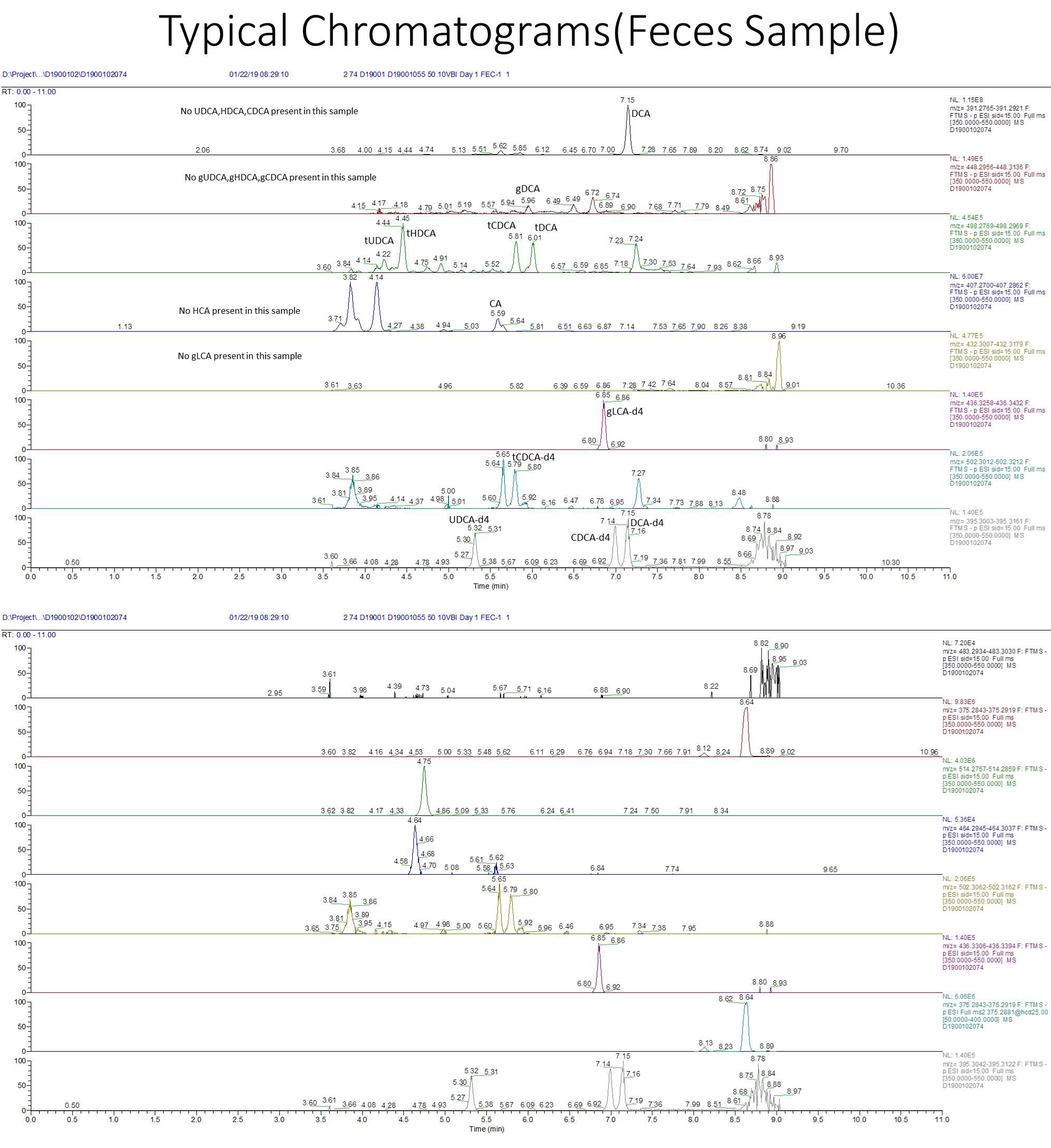

Supplement: Supplementary file 2 — Fig S2 [file FSN3-9-429-s002.jpg]
